# Fron-Mueller’s Case of Singular Formation of Cataract

**Published:** 1844-09-21

**Authors:** 


FRON-MUELLER'S CASE OF SINGULAR FORMATION OF CATARACT.

A merchant, aged 65, who had previously enjoyed good health, while sitting one day opposite a window was struck in the face by a sun-beam, and suddenly experienced severe pain in the right eye. The pain soon diminished; but vision, which was previously perfect, was quite lost on that side. He was seen three days after by Fron-Mueller, who discovered a lenticular cataract of the right eye. On examining the window through which the light passed, Fron-Mueller discovered, in one of the panes of glass, two convex bulbs, similar in size and form to lenses, a circumstance which probably explains the occurrence.—*Lond. and Ed. Monthly Jour.*, *from Gaz. Medica di Milano.*

